# Multi-Sensor Data Fusion Solutions for Blind and Visually Impaired: Research and Commercial Navigation Applications for Indoor and Outdoor Spaces

**DOI:** 10.3390/s23125411

**Published:** 2023-06-07

**Authors:** Paraskevi Theodorou, Kleomenis Tsiligkos, Apostolos Meliones

**Affiliations:** Department of Digital Systems, School of Information and Communication Technologies, University of Piraeus, 18534 Piraeus, Greece; ktsiligkos@unipi.gr (K.T.); meliones@unipi.gr (A.M.)

**Keywords:** sensor fusion techniques, deep learning, computer vision, assistive technologies, comparison analysis, remote sensing, usability and user experience

## Abstract

Several assistive technology solutions, targeting the group of Blind and Visually Impaired (BVI), have been proposed in the literature utilizing multi-sensor data fusion techniques. Furthermore, several commercial systems are currently being used in real-life scenarios by BVI individuals. However, given the rate by which new publications are made, the available review studies become quickly outdated. Moreover, there is no comparative study regarding the multi-sensor data fusion techniques between those found in the research literature and those being used in the commercial applications that many BVI individuals trust to complete their everyday activities. The objective of this study is to classify the available multi-sensor data fusion solutions found in the research literature and the commercial applications, conduct a comparative study between the most popular commercial applications (Blindsquare, Lazarillo, Ariadne GPS, Nav by ViaOpta, Seeing Assistant Move) regarding the supported features as well as compare the two most popular ones (Blindsquare and Lazarillo) with the BlindRouteVision application, developed by the authors, from the standpoint of Usability and User Experience (UX) through field testing. The literature review of sensor-fusion solutions highlights the trends of utilizing computer vision and deep learning techniques, the comparison of the commercial applications reveals their features, strengths, and weaknesses while Usability and UX demonstrate that BVI individuals are willing to sacrifice a wealth of features for more reliable navigation.

## 1. Introduction

Blind and Visually Impaired (BVI) individuals face a tremendous number of challenges even for simple daily routine tasks. One of those tasks is safe autonomous navigation in outdoor and indoor spaces. Enabling the BVI individuals to independently move around has a tremendous effect on several aspects of their mental and emotional wellbeing as it fosters a higher sense of empowerment and overall satisfaction by reducing feelings of dependency, isolation, and frustration that may result from consistently relying on others for mobility. Besides the above, autonomous navigation is critical to activities such as social participation, access to education and employment leading to the personal growth and development of the BVI individual.

The scientific community has proposed and demonstrated numerous solutions that span several different techniques and approaches to address these issues. However, few of them can be used immediately by the BVI individuals to improve their life as they are largely exploratory and most commonly have little to no consideration for the utility aspect of day-to-day application [1,2,3,4,5,6,7,8,9,10,11,12,13,14,15,16,17,18,19,20,21,22,23,24]. Furthermore, contrary to outdoor space navigation, which leverages the GPS infrastructure, indoor space navigation has no adequate solution. This was highlighted from discussions with both Orientation and Mobility specialists as well as BVI individuals on various occasions [25,26]. Simultaneously, there exist several commercial applications that are being used among the BVI communities. These include AriadneGPS [27], Lazarillo [28], Nav by ViaOpta [29], Seeing Assistant Move [30] and Blindsquare [31] among others with the latter being the most popular in the communities of the BVI.

Between these two there is a gap. On one hand, as can be seen from the literature, multimodal solutions can provide features that can improve the quality of life of BVI individuals. The abundance of sensors with varying capabilities and costs can be exploited with the help of sensor-fusion techniques with smartphone devices playing the role of the central point of integration. On the other hand, commercial applications already provide features to improve the experience of BVI individuals via the use of smartphones, but they could significantly increase their quality of service with the adoption of mature features found in the literature.

This paper, in an effort to close the gap, tries to provide a comprehensive review of the landscape of the available academic and commercial solutions. It performs a comparative analysis between these two to highlight the type of features that are part of commercial applications, the ones that are still part of the academic realm and the ones being the low-hanging fruits that can be immediately adopted from commercial applications. This effort strives to contribute to a dramatical user experience improvement.

For the case of conducting the sensor fusion solutions review the selected papers consisted mainly of peer-reviewed journals and conferences from the last five years to present the most recent advances. The scope of the search involved papers demonstrating sensor fusion solutions with an emphasis on applications for BVI individuals. The collected papers were evaluated from the standpoint of the solutions’ novelty, whether the employed procedure was adequately described as well as whether the proposed solution was thoroughly evaluated. For each paper, summaries were compiled and classified according to the chronological period, the sensor fusion techniques, the number and type of employed sensors as well as the practicality of the proposed solutions.

For the case of conducting the commercial comparative analysis, the selected applications were chosen based on the following criteria. The actual usage and popularity, as indicated by search engines, scores and critics as found in the smartphone stores (Play and Apple Store) and, finally, from interviews and questionnaires with BVI user communities. The evaluation of the commercial applications consisted of two parts. The first part concerned the comparison of the applications feature-wise while the second, their comparison from a Usability and User Experience (UX). For the latter part, the BVI individuals were requested to complete a UX questionnaire to quantitively assess their experience. At the same time the research team was monitoring the efforts of the participants in order to measure Effectiveness and Efficiency, the remaining two aspects of the Usability evaluation.

The paper’s content is organized as follows. The Materials and Method section presents to the reader the selection criteria of both the papers constituting part of the literature review and of the available commercial solutions in more detail. Furthermore, it describes the application comparison methodology. Followingly, the section Results demonstrates the findings of both literature review and comparative analysis. Finally, the conclusion-discussion section summarizes the findings and presents opportunities for improvements from the literature that can be immediately adopted by the existing or new commercial applications resulting in a significant improvement in user experience.

The study aims to link the state-of-the-art research on sensor fusion with the practitioners and developers of applications targeting BVI individuals. To the best of our knowledge, there is no other similar effort in the field. It does so by bringing together the most recent research literature with an exposition of their strong and weak points along with the most widely used international applications where a comparative analysis makes clear the number and type of features supported. Furthermore, there is no prior work presenting either a comprehensive list of features of the commercial applications or a Usability and UX study performed on commercial applications. Moreover, among the key takeaways of this study is a set of minimum features that outdoor and indoor blind navigation applications need to support along with improvements commercial applications could make by adopting features found in the research literature. The emphasis of the research study was on indoor space navigation as both O&M specialists and the individuals themselves admit both inadequate solutions and fragmentation of the space leading to limited awareness of the existing solutions.

## 2. Materials and Methods

This section presents the methodology for conducting both a literature review on papers employing sensor fusion techniques in applications targeting the BVI individuals and a comparative evaluation of the commercial applications consisting of two parts. The first part concerns the comparison of the applications feature-wise while the second concerns the comparison from the standpoint of usability of two of the most popular commercial applications, namely Blindsquare and Lazarillo, with BlindRouteVision, our outdoor navigation application.

### 2.1. Process of Conducting a Systematic Literature Review

**Figure 1:**
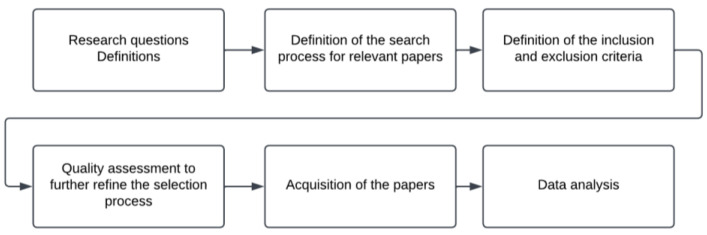
Reviewing Research Literature Methodology.

Critical to the conduct of any survey is the selection of a process to guarantee the validity, correctness, and effectiveness of the survey results. In particular, the adopted methodology (Figure 1) for conducting the systematic literature review [32,33] includes the following:

- Definition of the research questions
- Definition of the search process for relevant papers
- Definition of the inclusion and exclusion criteria
- Quality assessment to further refine the selection process
- Acquisition of the papers
- Data analysis

#### 2.1.1. Research Questions

Several research questions were defined to guide the review of sensor fusion solutions used for BVI applications and the commercial applications comparison.

Central to our approach was understanding the techniques employed in sensor fusion solutions for the designated application domain as well as uncovering the area’s trends, the frequency and the number of papers published. Details such as the type, characteristics and range of sensors used in these assistive technology solutions were also investigated. Finally, special attention was also given to the assessment from a practical standpoint, the cost and wearability efficiency to determine the degree to which they can be easily adopted and used long-term by the BVI individuals.

#### 2.1.2. Search Process, Inclusion, and Exclusion Criteria

After forming the research questions, the search process started. For the first task, various search engines and known repositories of scientific papers were selected as sources. The chosen papers consisted mainly of peer-reviewed journals and conferences where the ones from major publishers were prioritized, including IEEE Xplore, ACM Digital Library and MDPI among others. The scope of the search involved papers demonstrating sensor fusion solutions with an emphasis on applications for BVI individuals. This was applied by searching for a direct mention of the “sensor-fusion” and/or “navigation” term either in the abstract or inside the main text. Furthermore, at a later stage and as the previous search revealed a few results, we extended the search to cover implementations that are not part of assistive technology solutions for the BVI individuals but, nonetheless, their proposed solution could be transferred to our target domain. On the other hand, we excluded papers based on the following criteria:

- No mention of sensor fusion whatsoever
- Solutions that used similar techniques but with no application to our domain.
- Papers which mention sensor fusion as related work or use the phrase “sensor fusion” but do not elaborate on their sensor fusion procedure or they misleadingly use the term.
- Finally, papers that use sensor fusion, but their contribution is not very novel.

#### 2.1.3. Quality Assessment, Acquisition and Data Analysis

After gathering all the relevant papers, a second stage of filtering was applied so as to limit even further the number of papers and improve the quality of the study. In this stage, we considered further criteria including the solutions’ novelty, whether the employed procedure was adequately described as well as whether the proposed solution was thoroughly evaluated. After the quality assessment stage, the pool of papers was finalized, and the data analysis commenced. For each paper, summaries were compiled and classified according to the chronological period, the sensor fusion techniques, the number and type of employed sensors as well as the practicality of the proposed solutions.

### 2.2. Process of Conducting the Commercial Applications Review

**Figure 2:**
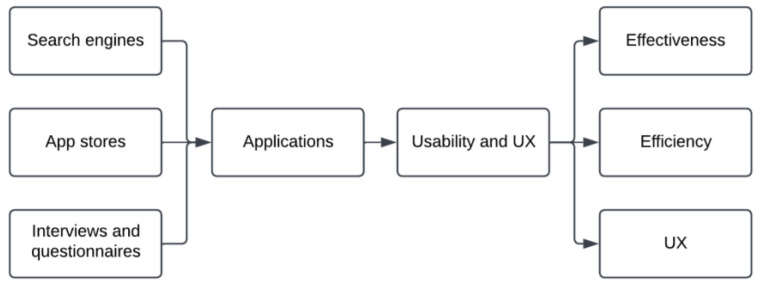
Commercial Applications Methodology.

One of the main goals of the commercial applications comparison was to gain an understanding of the employed techniques, the trends as well as the popularity of commercial applications among the BVI communities. Part of this effort was to also understand the advantages and disadvantages of those solutions’ features, weight and cost-wise. A crucial issue was to compare the effectiveness and efficiency of these solutions as well as their perceived Usability and UX with the help of BVI individuals. We, also, included BlindRouteVision, an application developed by our research team in the context of the MANTO project [34], to determine the effectiveness and efficiency of our approach as well. An emphasis was also given to uncovering the factors that make a successful commercial application for the BVI in which training is a crucial parameter and conventionally requires access to sites including special navigation utilities for the blind [35]. Finally, we conclude the presentation by highlighting the features found in the state-of-the-art literature that are easily transferred to the existing solutions. Figure 2 presents graphically the methodology employed to collect the commercial applications and evaluate them.

#### 2.2.1. Description of the Search Process

Likewise to the case of the literature review, the search of the commercial applications followed a process which was more simplified and less restrictive given the substantially smaller amount of available pool. In particular, the criteria that shaped the results were based on results from search engines, the popularity of the applications as indicated by the search engines, scores and critics of these applications as found in the smartphone stores (Play and Apple Store) and, finally, from interviews and questionnaires with BVI individuals concerning their experience with these applications as well as whether they use any of the available commercial applications as their main daily driver.

#### 2.2.2. Overview of the Comparative Evaluation of the Commercial Applications

The commercial applications’ comparison process was the following. Firstly, we downloaded them on compatible smartphone devices. Subsequently, the research team performed two tasks. The first one involved the applications assessment feature-wise which led to the compilation of a comparative table while the second involved the participation of the BVI individuals. The latter required them to navigate a set of preselected routes, one known and one unknown to them, to evaluate from a Usability standpoint the provided features of the two most popular commercial applications (Blindsquare and Lazarillo) and our application (BlindRouteVision) under a given set of criteria as defined by our research team. Then the BVI individuals were requested to complete a UX questionnaire to quantitively assess their experience. At the same time, the research team was monitoring the efforts of the participants in order to measure Effectiveness and Efficiency, the remaining two aspects of the Usability evaluation. The test subjects consisted of 13 BVI individuals both experts and non-experts with assistive technologies and with or without high digital sophistication. Both the Lighthouse for the Blind of Greece and the Tactual Museum of Athens helped to reach the participants.

#### 2.2.3. Usability and UX Evaluation

Usability and UX are two key terms in evaluating assistive technology applications for the BVI which are commonly overlooked and, thus, underexplored. These two have the potential to cast some light on the factors that lead to the low adoption rates of the BVI communities [36]. We have seen in the context of designing, implementing and evaluating our assistive technology applications that their exploration can lead to uncovering the system’s failing aspects from the perspective of the BVI individuals [37,38].

In order to assess the degree to which users can use them correctly to achieve their purpose while being satisfied at the same time, we employed three measures, namely effectiveness, efficiency and satisfaction. According to the ISO/IEC 25010 2011 [39], they are all components of usability. The latter is defined as “the degree to which a product or system can be used by specified users to achieve specified goals with effectiveness, efficiency and satisfaction in a specified context of use”. In particular, the 3 main components are defined as follows:

- Effectiveness—a measure of the degree to which users can complete a task.
- Efficiency—a measure of the time required by a user to complete a task.
- Satisfaction—a measure of the subjective quality of interaction with the application which is equivalent to UX [37].

#### 2.2.4. Effectiveness and Efficiency

The assessment methodology employed the metrics of effectiveness and efficiency and is similar to the one described in [37,38]. The former employs the metrics of completion and error rate and efficiency is measured as the ratio of successfully completed tasks per unit of time. These metrics are among the most used as they are simple and easy to understand, thus, making them popular in many studies. In particular, the Completion rate measures the number or percentage of tasks completed successfully, while the Error Rate indicates the number of errors per user. Common cases of errors include undesired outcomes arising as a result of poor interaction with the system interface or insufficient information, as well as mental errors where users are unable to comprehend the system options. This was measured by the research team as the users were executing the tasks given to them. For the calculation of effectiveness and efficiency, the following formulas were used:

(1) $$Effectiveness=\frac{total\;\#\;of\;tasks\;successfully\;completed}{total\;\#\;of\;tasks\;undertaken}=\frac{\sum_{l=1}^{U}\sum_{i=1}^{Μ}task_{li}}{U*M}$$

where *U* = # of participants, *M* = # of tasks per participant and $task_{li}$= *i*-th task of the *l*-th user. Furthermore, $task_{li}$ takes the value 1 if the task is successfully completed and 0 otherwise.

(2) $$Efficiency=\frac{\sum_{j=1}^{U}\sum_{i=1}^{M}tasks_{ij}t_{ij\;}}{\sum_{j=1}^{U}\sum_{i=1}^{M}t_{ij\;}}\times 100\%$$

where $t_{ij\;}=EndTime_{ij}-StartTime_{ij}$, for which, in turn, $EndTime_{ij}$ is defined as the time required for the *i*-th task of the *j*-th user to be completed successfully or the time until the user quits.

#### 2.2.5. User Experience—UX

User experience is usually measured with the deployment of questionnaires. To the best of our knowledge, there are no known questionnaires available that assess the features of assistive technology solutions targeting navigation for the BVI. The framework adopted for the purpose of this study is a modified version of the standardized UEQ+ framework [40]. The latter is a configurable, although limited, set of scales with the intent to quantitatively measure the opinion of a user on a Likert scale of 1 to 7 where the value 1 corresponds to “not at all satisfied” and the value 7 corresponds to “extremely satisfied.”

For our case, the set of included scales includes the following:

- Efficiency—assesses the users’ perception of the effort required to achieve the desired goal as well as how quickly the application reacts to their actions.
- Dependability—assesses the users’ perception of the system’s response predictability and consistency and the degree to which everything is under control.
- Personalization—assesses the users’ perception of the system’s configuration and adjustment to personal preferences.
- Usefulness—assesses the users’ perception of the gains as a result of using the system.
- Trustworthiness of content—assesses the users’ perception regarding the quality and reliability of the system’s instructions.
- Response behaviour—assesses the users’ perception of the utilized voice.

Finally, before handing out the questionnaire to the participants, they were requested to use the following evaluation criteria to form the basis of their qualitative assessment while navigating the suggested routes:

- Evaluation of the real-time tracking and navigation instructions
- Accuracy and density of reported position
- Combination with public means of transportation
- Traffic light crossings
- User Placement to Points of Interest (POIs)
- Obstacle detection and recognition

## 3. Results

This section presents the results of both the literature review on sensor fusion solutions for the BVI as well as the comparison of the commercial applications feature and usability-wise following the methodology described in Section 2.

### 3.1. Literature Review

The review consisted of 40 papers from various scientific and engineering societies as well as well-established publishers. The full list of the paper sources includes IEEE Xplore, ACM Digital Library and MDPI. The target application domain consisted of applications providing the tools for blind indoor and outdoor navigation with sensor fusion as the enabling technology. The selected papers ranged between 2018 and 2023 (the last five years).

**Figure 3:**
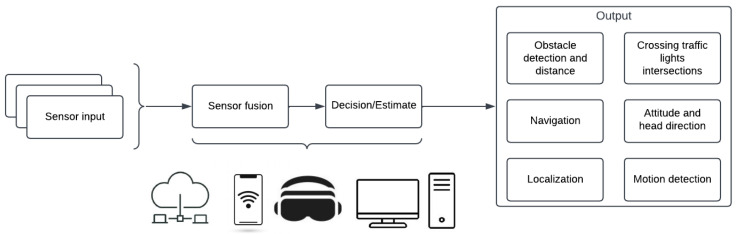
Commercial Applications Methodology.

The overall architecture of the proposed solutions is presented in Figure 3. Several sensors either homogeneous or heterogenous in nature feed the proposed sensor fusion systems. The majority of these sensor fusion systems run either on single devices, including smartphones and computer systems or in collaboration with the Cloud and/or other resources found in the near vicinity. Their goal is to make either a decision or to give an accurate estimate on various aspects associated with outdoor and indoor navigation.

Table 1 and Table 2 describe the sensor fusion techniques respectively and the type of employed sensors.

From the review, the following trends emerged. Most of the proposed solutions address aspects of indoor navigation with a percentage of 89% as opposed to 11% of the outdoor space solutions. Obstacle detection, a very important feature for BVI applications to possess, constitute 35% of the total while roughly half of them (46%) implement obstacle avoidance as well. The difference between these two is subtle but important. An obstacle avoidance solution, besides incorporating obstacle detection as a component, has the added feature of adjusting route navigation so as to provide the correct instructions to the users to avoid the obstacle. On the contrary, obstacle detection solutions go as far as detecting the obstacle while leaving the steps to bypass the detected obstacle to the user. Table 3 contains all the solutions of the literature review reporting, when available, whether they support obstacle detection, either static or dynamic or both, the supported range and accuracy.

The most preferred technique is the Kalman filter with a percentage of 27% followed by computer vision and deep learning approaches with the same percentage of 19%. The Kalman Filter, which is an algorithm used for estimation (correcting predictions), is favoured for its scalability. This Bayesian fusion method is particularly helpful for state propagation and updating basic input data. Next are solutions utilizing PDR with a percentage of 14% and Particle Filters with a percentage of 8%. The proposed solutions are multimodal, however, IMU sensors, a combination of accelerometer, gyroscope and magnetometer, are by far the most preferred (57%) followed by camera sensors (38%), Lidar sensors (16%), and, last but not least, ultrasonic sensors (14%). The prevalence of the IMU and camera sensors is expected given the proliferation of smartphone devices as the preferred platform for developing solutions for the BVI individuals. Finally, only 14% of these solutions can be used in real-life scenarios with some degree of success, 29% of them are practical but have a combination of either high cost or require from the user to carry many sensors, 24% are limited practicality for specific scenarios while 32% are purely experimental.

The following sections will present the solutions found in the literature organized around the sensors employed. Specifically, the presentation is organized around the criteria of being camera or non-camera assisted as different sets of techniques are required for those two cases. For each of these two categories, we continue by grouping the provided solutions based on the type of sensor with the most frequent group being presented first. For both cases, the sensor fusion techniques are described in a comprehensible manner.

In more detail, Section 3.1.1 covers the papers utilizing a form of the camera sensor, be it a single camera or a configuration of multiple cameras, headset devices as well as 3D camera sensors. Subsequently, papers utilizing the IMU sensor (accelerometer, gyroscope, magnetometer), the most popular sensor among the selected papers, are presented. For each individual paper, a summary incorporating the solution with the advantages and disadvantages is written. On the other hand, Section 3.1.2 concerns solutions with no camera sensors. Most of them incorporate an IMU sensor while other options include ultrasonic and Lidar sensors as well as Bluetooth Low Energy (BLE) beacons and Wi-Fi access points.

#### 3.1.1. Camera-Assisted Solutions

An uncertainty-based adaptive sensor fusion framework for Visual-Inertial Odometry (VIO) is proposed in [41] for estimating relative motion. It minimizes degradation from inaccurate state estimation by determining the states that should be included in the estimation process. These degrading states can arise under motion characteristics that nullify the readings of the inertial measurements such as when the user moves at a constant velocity. The level of uncertainty in the estimated states is measured using the expected value of the errors, which are calculated by reusing Jacobian matrices obtained from bundle adjustment.

A challenge of visual odometry concerns scenarios with scene illumination or featureless surfaces. In an effort to address the challenge [42] proposes a new deep-learning method to accurately estimate ego-motion using low-cost mmWave radar technology. They introduce a mixed attention approach to fuse the mmWave pose estimates with data from other sensors such as inertial or visual sensors, in order to improve the overall accuracy and robustness of the ego-motion estimation.

In order to improve the accuracy and robustness of monocular VIO in estimating the trajectory, the work in [43] proposes a framework that selectively fuses monocular images and inertial measurements. It is designed to handle real-life issues such as missing or corrupted data and poor sensor synchronization and includes two different feature selection strategies, namely deterministic soft fusion and stochastic hard fusion. These strategies are used to re-weight the concatenated inertial-visual features selectively, taking into account the current environment dynamics and the reliability of the data input.

The work in [44] proposes a localization technique that utilizes Progressive Sampling Consensus (PROSAC) and combines monocular visual and inertial navigation for improving the self-positioning of low-cost devices in unknown environments. The proposed solution for localization involves using a ROSAC mismatch culling approach that combines monocular vision and inertial navigation.

The system in [45] implements an attitude estimation method based on monocular vision and inertial sensor fusion for indoor navigation. It attempts to mitigate the low accuracy in the results of attitude estimation based on vision and inertial sensors due to factors such as motion image blur, attitude angle processing algorithms, and data synchronization. The main method of attaining the high-precision attitude information of both the visual and inertial model is a multi-rate Kalman filter. When a new image is collected by the camera module, the visual attitude measurement algorithm is used to solve the zero-bias error of the new gyroscope, and then the results of inertial measurement and visual measurement are combined to update the attitude angle information.

Detecting when the user is in an outdoor or indoor space is helpful. The system in [46] implements a pedestrian navigation methodology optimized for this case. To enhance the accuracy of the location identification, the navigation algorithm integrates signals from the light and magnetic sensor as well as from the global navigation satellite system (GNSS). In more detail, the information of the satellite and magnetic sensors are introduced into the extended Kalman filter data fusion processing to reduce the positioning error. The fusion of these sensor readings is done with the help of a decision table constructed to detect whether the device is in an outdoor or indoor space.

The work in [47] proposes a solution that focuses on navigation, localization and orientation of visually impaired individuals in outdoor and indoor spaces using a non-GPS approach. The main approach of the system is to jointly utilize dead-reckoning and computer vision techniques on a smartphone-centric tracking system. Input from the gyroscope and the accelerometer are fused with the camera module-generated information to create the heading estimate. The latter is computed by a prediction-correction filter.

Few efforts cover both indoor and outdoor navigation. A system describing an indoor and outdoor tracking and navigation system that uses pedestrian dead reckoning and computer vision algorithms for indoor environments and GPS for the outdoors is described in [48]. The system takes advantage of coloured tapes as reference signals to turn the smartphone camera into an additional sensor. By processing images from the camera, the system can estimate user heading and velocity, and detect landmarks to reset tracking errors. The accuracy of the system is improved by combining measurements from the smartphone’s IMU and computer vision techniques via the use of an Extended Kalman Filter.

Videos and inertial sensor fusion can be leveraged in systems providing real-time traffic light detection systems for BVI pedestrian navigation [49]. The inertial sensors are used to estimate the orientation, by fusing gyroscope, accelerometer and magnetic field sensor data, and motion of the camera, which is used to correct for the distortion in the image caused by the camera’s tilt and to calculate the position of the traffic light in the image. The position information is then fed into a Convolutional Neural Network (CNN) trained to recognize traffic lights, enabling the algorithm to detect traffic lights with greater accuracy. The paper also proposes using the velocity and acceleration estimates from the inertial sensors to predict the position of the traffic light in subsequent frames. This reduces the computational load required for real-time detection and the spurious detection of traffic lights.

The work in [50] proposes the design and implementation of a personal assistant robot for BVI individuals using sensor fusion technology for indoor navigation to provide accurate robot localization and navigation. The sensor fusion is responsible for fusing data from the camera and the lidar system to provide a map and the depth of the area. This is especially helpful in scenarios where the depth information obtained from the camera is degraded due to the configuration of the indoor space such as uniform wall colours.

Combining both the Cloud and the Edge can help in providing robust solutions for indoor navigation by raising situational awareness and providing navigational aid for BVI individuals. The solution in [51] fuses data generated from the smartphone’s LiDAR and camera sensor as well as applying machine learning algorithms that are partly performed in the cloud and partly in the edge. The system’s main functionality is to provide a rich 3-D description of the user’s front-facing navigational path by enhancing the camera-captured image of a scene with LiDAR-generated distance information and directional information computed by the device. Additionally, the system combines sensor data fusion and geometric formulas to generate step-by-step walking instructions for the user to reach the desired destination.

Another system [52] utilizing sensor fusion to address both indoor and outdoor space navigation consists of a navigation assistant and a wearable device with a camera. It combines computer vision and sensor-based technology, in a cost-efficient way, to detect multiple objects and enhance the accuracy of its collision avoidance system. The employed algorithm performing the fusion utilizes a fuzzy controller with 18 fuzzy rules that take as input the user position from the sensors and the distance of the detected obstacle via employing computer vision techniques on the camera module. As a result, it returns audio instructions as to what the user should do.

The work in [53] proposes a method that involves using two RealSense R200 (Intel, Santa Clara, CA, USA) devices to create a colour stereo vision system with a short baseline for improved depth estimation. As the depth map produced by the R200 is not accurate at short distances, a stereo matching algorithm that uses the colour stereo image pair to generate a more precise depth map was developed. This new colour stereo depth map is then fused with the original depth map created by the R200, which is produced through a stereo-matching process using the infrared image pair. The fusion algorithm works on each individual pixel. A voting window is set up around the pixel in question, and any other pixel within that window will vote based on similarity. The depth value that receives the most votes becomes the final depth value for the pixel.

Several systems employ an RGB-D camera as a central component. In [54] a system equipped with an RGB-D camera and ultrasonic sensor is used to navigate the BVI individuals. The proposed fusion method combines the distance measurements from the ultrasonic sensor, which correspond to the ground plane, with the depth measurements from the RGB-D camera by mapping the ultrasonic sensor’s distance readings onto the RGB-D camera’s point cloud. The fused data is processed using an extended Kalman filter (EKF) to estimate the user’s position and orientation. The EKF incorporates the sensor measurements, the user’s motion model, and an error model to estimate the user’s position and orientation over time.

Another system employing sensor fusion for assisting the navigation of the BVI individuals comprises an RGB-D camera with a millimeter wave radar [55]. It fuses data from those tow sensors to detect obstacles in the user’s navigational path. The system acquires data on the velocity of multiple objects via a frequency-modulated continuous wave millimeter wave radar and it performs contour extraction and applies MeanShift algorithms on the output of the RGB-D sensor to verify the obstacle’s position. The fusion of data from the millimeter wave radar and RGB-D sensor is achieved via the application of particle filters to obtain an accurate state estimation. Another benefit of this approach is its suitability for scenarios that have multivariate, non-linear behaviour and noise that follows a non-Gaussian noise. Following this approach successfully bypasses the complexity of applying the Joint Integrated Probabilistic Data Association (JIPDA) algorithm that is able to track and label multiple targets prior to the step of applying the Kalman Filter.

The work in [56] is proposing a multi-sensor fusion system for improving indoor mobility of the visually impaired in corridor environments. The multi-sensor system employs floor-plan digitization, semantic SLAM, environmental perception, obstacle avoidance and human-machine interaction modules. The system improves its results by fusing environmental data and depth information. The environmental perception data is produced by combining RGB-D and Lidar sensor data.

A system for exploring and navigating areas with BVI individuals that is both safe and adaptable to changing environments and runs in real-time is presented in [57]. The system leverages the computational capabilities of the iPhone and ARKit for obstacle detection, using a combination of 2D object detection, semantic segmentation, 2D object tracking, 3D ARKit point cloud, and depth maps generated by LiDAR sensors in recent iPhone versions. In particular, the system fuses the generated input from the camera and LiDAR sensor of the iPhone. The method also uses the plane detection capability of ARKit to detect objects with planar surfaces, this covers large objects that cannot be detected by the 2D object detector.

An indoor obstacle detector in combination with a navigation module to assist in real-time BVI individuals is presented in [58]. In particular, the navigational work as well as the obstacle detection portion was carried out by the combined functioning of the PIR Sensor and the Ultrasonic Sensor which are connected to the Arduino Nano processor.

Detecting and identifying obstacles along the navigational path of a BVI individual is important. The system in [59] fuses the input of ultrasonic, vision and sonar sensors. The first two are used for obstacle detection and identification, respectively, while the last one speeds up the visual data processing as it allows the selection of only the regions which have an obstacle. The main approach concerning fusion is to use the Extended Kalman filter to fuse input from homogeneous sensors and rule-based fusion for data coming from heterogeneous sensors. Furthermore, camera rotations, which occur due to the body movements, are corrected by fusing the inertial measurement unit data connected to the camera.

#### 3.1.2. Non-Camera Assisted Solutions

The design and development of a wearable assistive device integrating a fuzzy decision support system, for the navigation of BVI individuals, is presented in [60]. The system consists of an acquisition system that takes input readings from the IMU and Mini-LIDAR sensor. By fusing the inputs from the IMU sensors using an Extended Kalman Filter (EKF) method data on attitude and head direction is collected while the fusion of velocity and depth data happens at the fuzzy controller to assess the level of risk on a given path during navigation.

The solution in [61] proposes an indoor positioning framework for BVI individuals using the Internet of Things (IoT). The system leverages the smartphone’s inertial sensors and Bluetooth-powered beacons in order to provide turn-by-turn instructions to a pedestrian to navigate between two points even in cases where external sensing is absent as in the case of a big hallway. The solution calculates the user’s threshold and step length to determine the travelled distance while these values are regularly updated for a user’s profile and stored in the cloud. Subsequently, the framework fuses the data generated from the travelled distance, heading and turns to estimate the current position of a user with the absolute location as it is inferred from the Bluetooth beacons.

Another approach to supporting an indoor position system is with the help of Wi-Fi RSSI trilateration and INS sensor system simulation. The system [62] employs a Kalman Filter to fuse the RSSI signals with the INS data to have more accurate positioning. The IMU acceleration is integrated to determine the INS position.

Reliable and accurate indoor orientation and localization can be achieved by the combination of IMU and magnetic sensors. An improved KF (Kalman filter) is designed [63] to combine data from those two sensors to achieve precise position and orientation estimation. The magnetic sensors are utilized to counterbalance the accumulated error and drift of the inertial sensors, while the inertial sensors are used to rectify errors in the magnetic fields that are related to orientation. Additionally, a parameter derived from the magnetic tensor is used to facilitate indoor obstacle avoidance and object/destination approach, as well as to indicate the reliability of the yaw angle estimated from the magnetic measurement.

To address the inaccuracy of the GNS navigation systems, a system detecting obstacles and estimating the user’s distance from them utilizing infrared sensors is proposed in [64]. It consists of a base station, equipped with a Bluetooth module and a microcontroller, receiving sensor data from a wearable device, equipped with IMU and infrared sensors, and performing sensor fusion to estimate the user’s position and orientation. The results are then transmitted back to the wearable device translated into haptic feedback so as to guide the user through the environment.

The work in [65] proposes a deep learning-based solution that aims to achieve robust inertial motion tracking in various environments. It incorporates additional IMU sensors, like smart earbuds, that are less likely to be affected by motion noise than the smartphone’s IMU measurements. Specifically, the fusion layer is a fully connected layer in the CNN that takes as input the features from the accelerometer and gyroscope sensors in both the smart earbuds and the smartphone. In general, the proposed sensor fusion model aims to synthesize the estimations from multiple sensors and automatically adjust their fusion weights according to their motion noise levels, in order to mitigate the impacts of the corrupted sensor. The fusion model assesses the reliability of the sensors based on a customized reliability LSTM (r-LSTM) and fuses the translation and angle increment features using two attention models.

The work in [66] proposes a solution for achieving localization accurately, continuously and in real-time utilizing a probabilistic localization algorithm running on a smartphone device. The algorithm takes advantage of the inertial sensors and the Received Signal Strength (RSS) from BLE beacons and tries to resolve many of the issues existing localization approaches face when deploying them in large and complex environments, like shopping malls and hospitals. The probabilistic framework uses the Particle Filter for state estimation.

A sensor data acquisition system employing multimodal sensor fusion for recognizing human activity using deep learning is presented in [67]. In addition to accelerometer data, gyroscope and magnetometer sensor data are collected and considered for multimodal sensor fusion to improve activity recognition performance. A classifier-level sensor fusion technique using a two-level ensemble model is adopted to combine class-probabilities from multiple sensor modalities, leading to an improvement in classification performance. The accuracy of each sensor on different activity types is analyzed, and custom weights are elaborated for multimodal sensor fusion, taking into account the characteristics of individual activities.

Another work [68] presents the design of a tactile display for BVI individuals to access virtual diagrams. The system utilizes a compact robot base with Omni-wheels that enables seamless and unrestricted movements in a two-dimensional space. The proposed sensor fusion approach uses a single optical mouse sensor and a commercially available IMU, without the need for placement away from the center of the robot base. The displacement data from the mouse sensor is combined with the orientation angle obtained from the IMU to determine the precise x and y location and orientation of the device.

A comprehensive deep learning approach to acquire meaningful insights from a variety of sensory data types and enhance the accuracy of classification and recognition tasks is presented in [69]. This framework merges unique and complementary information from multiple sensors and prioritizes high-quality data while also considering the relationships between different sensors. It utilizes both weighted-combination features and cross-sensor features to accomplish this goal.

The work in [70] proposes a solution that applies deep learning thermal-inertial odometry with visual hallucination. In particular, the proposed approach utilizes fusion to selectively combine features extracted from three distinct modalities, including thermal, hallucination, and inertial features to improve pose regression. By incorporating selective fusion into the combined features, the authors observe a consistent reduction in ATE compared to the network without selective fusion. This highlights the importance of selective fusion in achieving accurate results as each feature modality has its own inherent noise and the hallucination network may produce incorrect visual features.

The system in [71] supports indoor localization targeting BVI individuals by fusing a metaheuristic algorithm with a Neural Network using energy-efficient wireless sensor networks. To optimize the performance of the Artificial Neural Network (ANN) and improve localization accuracy, the authors integrated the ANN with the following six different metaheuristic algorithms: the backtracking search algorithm (BSA), the crow search algorithm (CSA), the gravitational search algorithm (GSA), slime mould algorithm (SMA), the particle swarm optimization (PSO), and the multiverse optimizer-ANN (MVO). Each algorithm was applied independently to determine the optimal number of neurons and learning rate for the ANN. As a result of this fusion, the authors were able to enhance the performance of the ANN and achieve a reduction in localization errors.

The design and implementation of an electronic aid for BVI individuals are presented in [72]. The system complements the white cane and uses a range of ultrasonic sensors, among others, in order to detect changes in motion and potential obstacles in the BVI individuals’ navigational path. In more detail, the system uses 6 ultrasonic sensors installed on a boot worn by the user and fuses utilizing a set of derived formulas to detect the events of ascending staircase, floor-level obstacles, knee-level obstacles and knee-level forward slanting obstacles.

Sensors available in commodity smartphones can be used to provide accurate and robust floor localization [73]. This is done by fusing Wi-Fi and barometer sensors in a hybrid probabilistic framework, allowing for plug-n-play floor localization without requiring prior calibration or extra geo-information that is inherent in fingerprinting. The latter is achieved by automatically crowd-sourcing the construction of histograms reflecting the probable locations of installed Wi-Fi access points (APs) in building floors, leveraging pressure readings.

Depth mapping with light or radio frequency for reliable indoor space positioning and multiple obstacle distance information have limitations due to the noise level, calculation complexity, reaction time and many others. To address these, the system in [74] consisting of a device using a single ultrasound source and two to three receivers attached to a headset employes ultrasonic sensor fusion to find obstacles. The results are then transmitted to the user via audio feedback.

A self-attention deep learning framework for leveraging the heterogeneous sensors of low-end IoT devices to improve the accuracy, granularity and amount of information is presented in [75]. The framework automatically balances the contributions of multiple sensor inputs over time by exploiting their sensing qualities. Firstly, it employs a self-attention mechanism to learn correlations between sensors, without the need for additional supervision, to determine the sensing qualities and adjust the model concentrations for multiple sensors over time. Secondly, instead of directly learning the sensing qualities and contributions, the framework generates residual concentrations that deviate from equal contributions, which helps to improve the stability of the training process.

Indoor navigation systems based on smartphones frequently utilize PDR coupled with an external source to correct the accumulated error. The works in [76,77] follow the abovementioned approach by combining PDR with BLE beacons and UWB inferred wireless position respectively. Both are fusing the inputs via an improved Particle Filter. The former approach constrains the movement of particles using a floor-map and updates the particle weights based on proximity to BLE beacons. This real-time localization method yielded promising results, without the need for a site survey when compared to fingerprinting localization. The latter approach involves a fusion filtering process that corrects the PDR-calculated positioning trajectory using the UWB positioning. This improves the accuracy and stability of the fusion positioning. A more simplified approach to improve the accuracy of indoor pedestrian localization has been adopted from [78] where the system takes input from inertial sensors, a light sensor, a smartphone-integrated Bluetooth-sensor and a digital map representation of the indoor space. Position information from the light sensor and Bluetooth is used to modify the step length and heading information, the user’s step length is modified by the distance between two ceiling-mounted lamps while the heading direction can be reset by the known planning route from the map. This information is leveraged to reduce the accumulated error as a result of the PDR method.

A unified fusion framework for indoor navigation addressing is presented in [79]. It can process various types of inputs, including Wi-Fi, Bluetooth, visible light, and images with geomagnetic sequences or Wi-Fi fingerprints. The framework uses convolutional or recurrent networks to extract initial features from the inputs while additional fully connected layers are inserted to map the features to a common space for effective fusion. Ensemble learning is used to reduce the impact of noisy signals on location and orientation estimations. Multiple models are trained using the training data, and their outputs are combined for fine-grained estimations. Mixture density networks are also incorporated to generalize the models with a mixture of distributions.

Finally, the work in [80] presents an indoor space navigation solution based on a wearable device providing BVI individuals audio assistance while traversing a navigational route equipped with markers. The sensor fusion approach utilizes visual and radio frequency markers to create a unique and consistent representation of indoor space. A Kalman filter is used to fuse the two data groups coming from the process of fingerprinting the internal space and from the visual markers. An LSTM network predicts the results of each sensor modality including an accelerometer, gyroscope and magnetometer which are used as an indication of the classification of probabilities of each activity class and are accepted as meta-features.

### 3.2. Commercial Review—Applications

The search for the commercial applications according to the search criteria defined in Section 2 resulted in the following applications:

- Blindsquare
- Lazarillo
- Ariadne GPS
- Nav by ViaOpta
- Seeing Assistant Move

BlindSquare [31] is a mobile application designed for blind, deafblind and partially sighted offering location-based information and navigation in a user-friendly way. It covers both indoor and outdoor navigation. The user selects a destination via a screen reader-friendly interface. Its support of GPS provides users with accurate and reliable information about their environment, such as nearby landmarks, road intersections, and POIs such as shops, public spaces, churches and many others. With the help of BLE beacons, BlindSquare provides accurate guidance information for indoor spaces. Furthermore, it offers the capability to personalize the application with information filters preventing information overload and, thus, enhancing the user experience. The information generated by the application is emitted via voiceover. Besides supporting cities, over the years BlindSquare has extended its coverage to other spaces including parks. Despite its proven robustness, its reliance on external services, like Foursquare and OpenStreetMap among others, can potentially deteriorate the provided quality of service when these are not reachable. Moreover, BlindSquare being a paid app available only on iPhone devices may be a barrier for some users, particularly those on a limited budget. In conclusion, despite some limitations, BlindSquare is a highly effective and valuable resource for individuals with visual impairments. Its numerous benefits, such as accessibility, customization options, and accurate location data, as well as its proven robustness over the course of years, outweigh any potential drawbacks, thus constituting the most preferable application by the BVI individuals. In the near future, BlindSquare intends to provide a set of new features and improvements [81]. These include enhancements to intersection-related information such as intersection clusters, travel direction, whether the intersection is surface or is an over/underpass, and other tips to validate travel. Another upcoming feature is object detection, especially, the ones which could cause an injury to the user. These objects will include stairways, picnic tables, benches, walks/sidewalks, bollards, gates and many others.

Lazarillo [28] is similarly a mobile application designed to assist BVI individuals in navigating both indoor and outdoor spaces. It provides audio instructions to guide users through physical spaces such as buildings or city streets. The app uses GPS technology to determine the user’s location and then delivers step-by-step directions through the user’s smartphone speakers or a connected headset. Via the use of BLE beacons and custom-made digital maps, Lazarillo can provide indoor navigation as well. The app is customizable, allowing users to adjust the volume and speed of the instructions, select a preferred language, and the types of alerts they receive. Moreover, the app has a user-friendly interface, and it is easy to use, even for people who are not technically proficient. Last but not least, Lazarillo is available for both Android and iOS devices. Overall, Lazarillo is usually preferred by the BVI individuals who own Android devices as highlighted in the interviews.

Ariadne GPS [27] is yet another mobile application designed to assist BVI individuals in navigating outdoor spaces. It provides audio instructions and feedback to help users navigate unfamiliar environments, locate POIs, and travel from one location to another. The app uses GPS technology to determine the user’s location while the guidance instructions are delivered through the user’s smartphone speakers or a connected headset. Ariadne GPS can be customized to meet the specific needs of individual users, including adjusting the volume and speed of the instructions, selecting a preferred language, and customizing the types of alerts they receive. In effect, Ariadne GPS has three primary functions: (1) Situational awareness—The application can provide information about the user’s location at any given time or automatically at predetermined intervals. The amount and detail of information can be either incremental for the automatic case or a full description when the user makes a request, (2) Favorites—Ariadne GPS allows the user to save favourite points, including bus stops, train stations, shops, and house doors, and receive alerts when approaching them, (3) Map exploration—The application’s map exploration allows users to touch with their finger any location on the map displayed such as a city, street, or country, depending on the screen’s zoom level, and the application will announce the name of that object.

Nav by ViaOpta [29] is designed to assist BVI individuals in navigating outdoor spaces and is part of the larger ViaOpta suite of mobile applications and services developed by Novartis, a multinational pharmaceutical company. The application provides voice-guided turn-by-turn directions, haptic feedback, and real-time location tracking to help users navigate safely and efficiently. The application’s user interface is designed to be simple and easy to use, with large buttons and text that are easy to read. It also offers features such as route planning, POIs, and the ability to save favourite locations. Furthermore, it informs users of intersections in close proximity along with their distances. It, also, enables the users to insert waypoints on routes so as to be closer to their liking. Nav by ViaOpta is generally fast and responsive, with minimal lag or delay in providing turn-by-turn directions and other information. The application also uses encryption to protect user data in transit and on its utilized storage service. Finally, the application is compatible with both iOS and Android mobile devices and is available in several languages, including English, Spanish, German, and French.

Last but not least, Seeing Assistant Move [30] is another mobile application with the purpose of enabling turn-by-turn outdoor navigation for BVI individuals. This application is particularly easy and intuitive to use due to its support of voice commands, allowing one to access its functions immediately. The app makes use of GPS technology that allows it to track the user’s movements in real-time and provides information on nearby POIs. Users can either determine the route to their destination by inserting points or leave the application to automatically determine one. Furthermore, it allows importing points of routes stored on databases supported by other systems. The application provides a route monitoring feature in order to easily return to user-specified points as well as a simulation of locations so as to familiarize with the area prior to starting the navigation. Moreover, the app also has a magnification function that allows users to zoom in on specific areas of the screen for a clearer view of the details. In terms of accessibility, the app is designed to be user-friendly for people with visual impairments or blindness, featuring a straightforward interface with large, readable buttons and text. Finally, the app has received awards and recommendations from the Polish Blind Association for its functionality, it is compatible with both iOS and Android mobile devices and is available for free download from both stores.

#### 3.2.1. Comparative Analysis—Features

All these applications support several features. Some of these are found on all of them to an extent, while others are exclusive to certain applications. Below, the comparative analysis of the supported features follows. Table 4 presents the total number of features that are supported by the set of selected applications and marks those that are supported per application.

The solutions explored are all provided for smartphone devices. The majority of them, including Lazarillo, Seeing Assistant Move and Nav by ViaOpta support both Android and iOS platforms, while Blindsquare and Ariadne GPS are found exclusively on iOS. The above table, also, indicates that Blindsquare is the solution with the most supported features, followed by Lazarillo, Nav by ViaOpta, and AriadneGPS with Seeing Assistant Move occupying the last position. All applications support outdoor navigation, route planning, real-time updates, report the user’s location either during the navigation or when the user requests to receive an update on the current location, support searching and sharing of places and POIs both in the near vicinity and in the wider area and, finally, are customizable each to its extent.

Exclusivity-wise, Lazarillo is the only application that supports the creation of digital maps and vehicle mode. With the former, Lazarillo can overlay information over floor plans, enabling businesses to participate and make themselves more accessible to the BVI individuals and become more involved, while the latter allows for the detection of speed changes in order to support the switch from pedestrian to vehicle mode and accommodate that use case as well. The application Nav by Opta, developed by Novartis, exclusively provides the feature to call for a sighted volunteer’s help to offer live support for emergencies. Blindsquare sets itself apart as it supports the largest number of unique features including (1) a multi-category search of places and POIs, (2) progress updates in audio message format with increasing frequency as the user approaches the designated destination, (3) an adjustable radius for the POIs, (4) support for the deafblind, (5) multi-input methods including the on-screen iOS keyboard, the iOS handwriting feature, dictating text to Siri as well as a 3rd party app that allows the user to use Braille, (6) audio menu where the user can control the Blindsquare from the headset and, finally, (7) providing a simulation tool of routes for raising the user’s awareness of the surrounding environment prior to actually traversing that route. The latter leverages Blindsquare’s Look Around feature, which is used to plan the trip ahead of time in order to increase the user’s confidence and sense of safety.

Examining the full set of exclusive features, a few of them can be considered useful to have for the provision of an increased level of quality of services. A quite useful feature for all the applications to have concerns the capability to create digital maps of indoors spaces of existing buildings, as provided by Lazarillo. This gives the opportunity for more detailed representations of buildings, thus allowing better and more secure indoor navigation. Furthermore, Lazarillo’s approach to bringing the business or the place in the loop is important from a scalability standpoint. In this way, the workload can be decreased and as such no single organization responsible for performing those actions can become the bottleneck in this process. Nav by Via Opta supports another interesting and very helpful feature as it allows for a BVI to call a sighted volunteer to provide support. This can be exceptionally helpful for cases of emergencies such as when a BVI is lost and cannot find another person to point them in the correct direction as well as when the BVI has been involved in an accident. On the other hand, all the features of Blindsquare are important in order to provide an increased level of quality of service for the BVI.

A very popular solution regarding the support of navigational information for routes, POIs, intersections and other static objects such as trees, dumpsters, benches and the like is OpenStreetMap. The latter is an open and freely available geographic database maintained by a community of volunteers via open collaboration and is used by all the selected applications but Ariadne GPS. Given this database stores a wealth of detailed information about an area, it is a very good solution for providing enriched semantic information. However, the downside to this is the possibility that an area’s info may not be accurate or even present on OpenStreetMap as the stored information depends on volunteers’ actions.

Another popular 3rd party service used by both Blindsquare and Lazarillo is Foursquare. The latter service provides the user with the capability to search for POIs including restaurants, cinemas, coffee shops and the like. Since this is a large database its wealth of information can be leveraged to provide a rich experience. Similarly to the case of OpenStreetMap, the downside to this solution is the dependence on a single service and the occasional deterioration of accuracy or complete lack of information.

#### 3.2.2. Effectiveness and Efficiency

Given the quite high number of applications, including our own, and the time-consuming aspect of the tasks, some of the participants expressed their concerns about their availability. To address this issue, we decided to proceed with the Usability and UX evaluation of two out of the five commercial applications and our own. Specifically, we evaluated Blindsquare and Lazarillo, which are the most popular, and BlindRouteVision.

After gathering the required data from the trials, we calculated the two metrics. Their results are presented in Table 5.

The results on Effectiveness show that our application was more favourably evaluated (80.56%) over the other commercial applications with Blindsquare being very close (78%). Lazarillo received the lowest score (66.6%) among the three with the difference from the top two being significant. Likewise, the Efficiency results follow the same trend. Our application is evaluated higher than the rest of the applications (78.12%). In the second place, we find Blindsquare (73%) while the last position is taken by Lazarillo (62%) with a significant gap to the other two again.

By analyzing and interpreting the results, the following conclusions can be reached. The participants favoured both BlindRouteVsion and Blindsquare almost equally, as their score indicates, while Lazarillo was the least favoured. Furthermore, a similar trend of Effectiveness and Efficiency is expected as they are closely related to the latter depending on the former. From our point of view, which was later confirmed as well as from the blind participants, the marginally higher preference for our application over the commercially available Blindsquare was due to the provision of more accurate navigation and, subsequently, the timing of events. This can be attributed to the use of the external GPS receiver from our application enabling more accurate and higher density tracking of the user location, coupled with our custom route navigation algorithm. Despite the wealth of Blindsquare’s useful features, as admitted by the blind users while justifying their reasons for preferring this application for their everyday activities, it utilizes the smartphone’s integrated GPS receiver, which is known to have a precision of fewer than 10 m and, thus, incorrect location reporting is frequent. In contrast, our application achieves an error of less than 1 m, thus, resulting in more precise navigation around sections critical to the safety of the BVI individuals such as corners and intersections giving users more control over the uncertain factors.

Lazarillo, on the other hand, scored low on both metrics as the application’s voice instruction subsystem significantly disrupted the blind users’ navigation with no control of this behaviour after the navigation process had started. The issue was occurring at intersections where the voice instruction subsystem would start spelling the names of the roads overwhelming any other information. This could be due to the names of the roads as the navigation took place in Greece or even specific to the operating system as Lazarillo was evaluated on Android. Despite the cause of this issue, blind users during the tests would have to stop as it was impossible for them to move forward. After some time, it started to become annoying for them and many requested to stop the task.

#### 3.2.3. User Experience—UX (Satisfaction)

This section presents the statistical results from the evaluation of BlindRouteVision, Blindsquare and Lazarillo. Various statistics were used and detailed reports were generated including the mean value with error bars showing the 95% confidence interval of the questionnaire’s scales, the consistency of the responses as measured with the help of Cronbach alpha, and, finally, the overall mean value per application of all the participants for evaluating the UX impression.

The supplied scales were evaluated using a Likert scale ranging from 1 to 7, however, their results were rescaled to the range of −3 to 3 so as to allow the comparison of the results with the initial version of the UEQ questionnaire.

**Figure 4:**
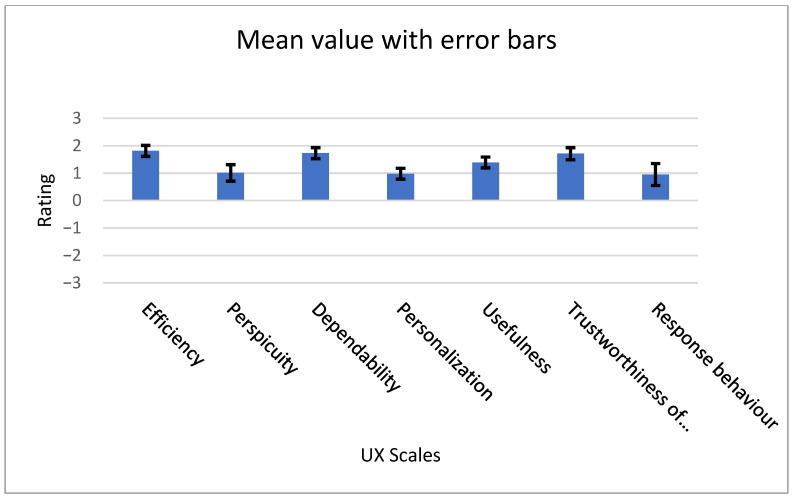
Mean values of scales—BlindRouteVision.

Starting from our own application BlindRouteVision, it can be seen from Figure 4 that it has been ranked high on the scales of Efficiency and Dependability with a score of 1.81 and 1.73 respectively. Close to those two is the scale of Trustworthiness of Content with a score of 1.71 while the scale of Usefulness received a score of 1.39. The high scores of these scales are in line with the observations made for the effectiveness and efficiency metrics and can be attributed to the highly accurate navigation due to the combination of the external GPS receiver and our custom navigation routing algorithm. The slightly reduced rating for the scale of Usefulness is due to the application’s lack of wealth of features that the other two commercial applications provide. The rest of the scales received mediocre ratings with Perspicuity, Personalization and Response Behavior having scores of 1.01, 0.98, and 0.95 respectively. These marginally positive assessments highlight future improvements for our application. Overall, the variability of the responses is low as suggested by the error bars in Figure 4 with the scale of Response Behavior having the largest observed (0.4) as there were varying opinions.

**Figure 5:**
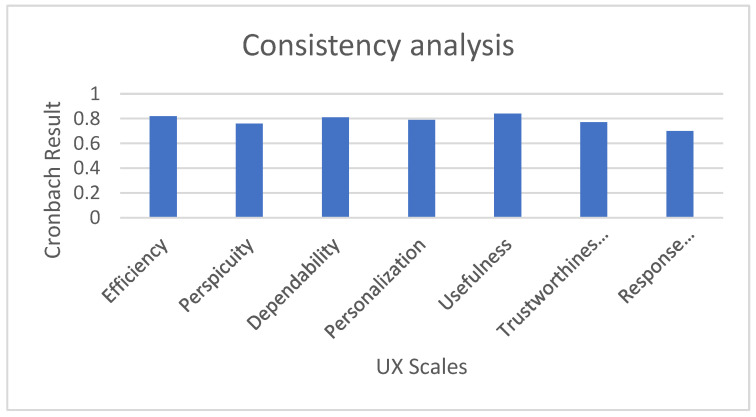
Consistency of scales—BlindRouteVision.

Figure 5 shows a bar graph of the consistency results as measured with the help of the Cronbach alpha coefficient. This metric is used to determine the reliability of the user responses. Although there is no generally accepted rule of thumb on the value the coefficient should have, however, in practice, a value greater than 0.7 is sufficient to qualify the results as reliable. As can be seen from Figure 5, the results are reliable.

**Figure 6:**
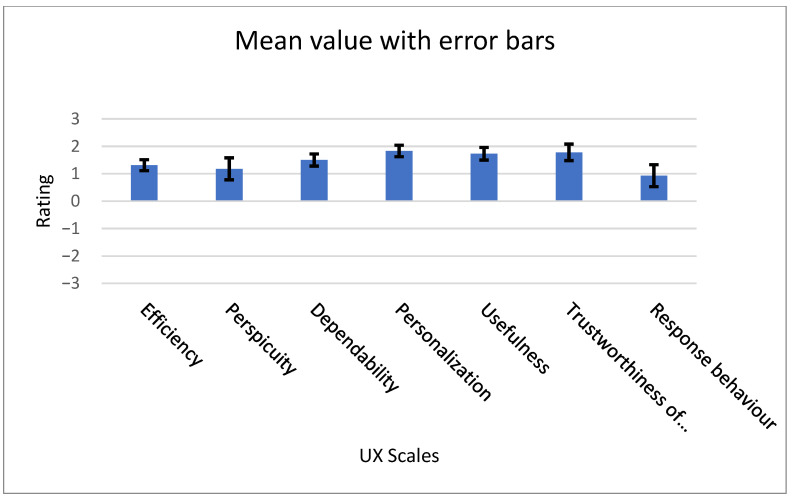
Mean values of scales—Blindsquare.

Next follows the Blindsquare application, the first of the two commercials. Figure 6 shows the overall evaluation as being a very positive one. Starting in reverse, the scale of Response Behavior has the lowest ranking with a score of 0.93. This can be seen as an area of potential improvement for the application as it concerns the qualitative elements of the voice characteristics emitting the information. The highest-ranking scale was Personalization with a score of 1.83 followed by Trustworthiness of Content and Usefulness with a score of 1.78 and 1.73 respectively. All these high assessments are due to the wealth of customizability and the accuracy of the provided information. The rest of the scales also have positive evaluations with a score of 1.5 for the scale of Dependability, a score of 1.31 for the scale of Efficiency and a score of 1.18 for the scale of Perspicuity. Overall, the variability of the responses is low as suggested by the error bars in Figure 6 with the scale of Response Behavior having the largest observed (0.4) as there were varying opinions.

**Figure 7:**
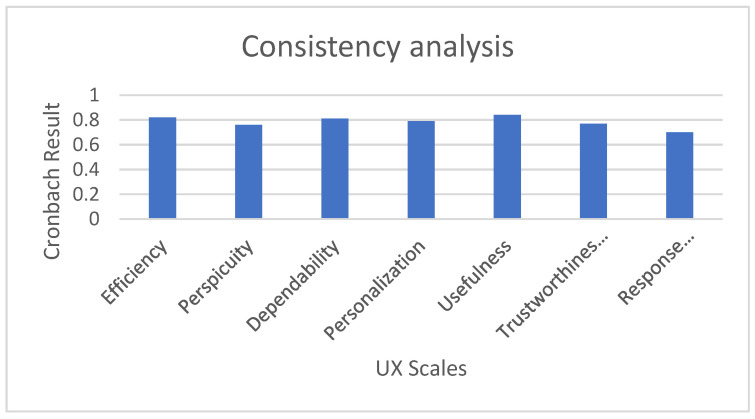
Consistency of scales—Blindsquare.

Figure 7 shows a bar graph of the consistency results as measured with the help of the Cronbach alpha coefficient. As can be seen, the results are reliable.

**Figure 8:**
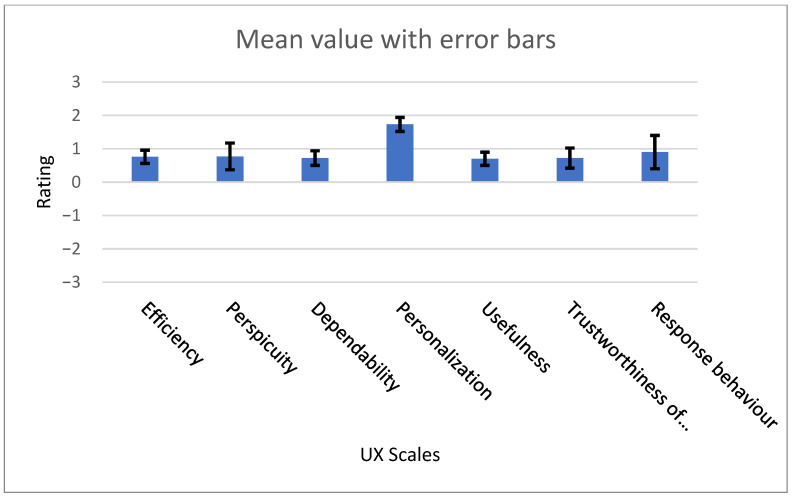
Mean values of scales—Lazarillo.

Last but not least, Figure 8 shows the evaluation of the Lazarillo application, the second commercial application. Among the three applications, it received the lowest positive evaluation. The major factor negatively impacting this application’s evaluation is the disruption caused to the navigation by the spelling of the roads at intersections as mentioned above in the section describing the evaluation of the effectiveness and efficiency metrics. The scales of Efficiency, Perspicuity, Dependability, Usefulness, Trustworthiness of Content and Response behaviour received scores of 0.76, 0.77, 0.72, 0.70, 0.72 and 0.9 respectively. The scale of Personalization is the only one that receives a very high positive evaluation with a score of 1.73. Lazarillo provides several customizable features that all blind users admit to be very useful. However, this alone is not enough to change the marginal positive evaluation. Likewise to the previous applications, the overall variability of the responses is low as suggested from the error bars in Figure 8 with the scale of Response Behavior having the largest observed (0.5) as there were varying opinions.

**Figure 9:**
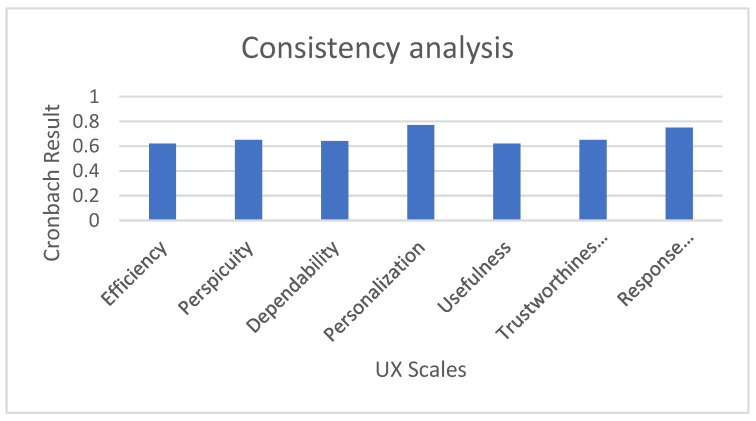
Consistency of scales—Lazarillo.

Figure 9 shows a bar graph of the consistency results as measured with the help of the Cronbach alpha coefficient. As can be seen, the results are reliable.

Overall, from the standpoint of UX, all of the applications received a positive evaluation. The highest was received by Blindsquare with a rate of 1.46 while the lowest one by Lazarillo with a rate of 0.9. The second place was occupied by our own application BlindRouteVision with a rate of 1.36. Despite showing the best results in the scales of Efficiency and Dependability, the lack of the wealth of features found in the commercial applications reduced its overall rate. Blindsquare received the best score for the scale of Personalization followed closely by Lazarillo. Common to all of the applications is the low score for the scale of Response behaviour indicating a low satisfaction with the voice instruction subsystem. All of them utilize the default voice systems provided by the platforms supported, which are not particularly well accepted by several blind participants.

## 4. Conclusions–Discussion

This paper had a twofold target. The first one concerned the conduct of a literature review concerning the use of sensor fusion techniques in blind navigation applications over the last five years. The second concerned the conduct of a comparative evaluation of commercial applications popular among the BVI communities. The scope of the evaluation concerned a feature-wise comparison of all the commercial applications and a Usability assessment including the two most popular applications among the BVI in Greece, namely Blindsquare and Lazarillo, with our own application BlindRouteVision.

For the first target, the main point of interest was the description of the fusion techniques used in applications targeting both outdoor and indoor space navigation for BVI individuals. The study involved the classification of the solutions in terms of the employed technique, the number and range of types of sensors, and their practicality regarding cost and wearability efficiency. The results demonstrated a limited number of solutions but with an upward trend in the number of available publications as the years progressed. Overall, most of the reviewed solutions concerned overwhelmingly the case of indoor space navigation covering 89% of the total amount. Out of the total number of applications, 35% of them supported obstacle detection while roughly half of them 46% implemented obstacle avoidance as well. Kalman Filter, computer vision and deep learning approaches were heavily utilized accounting for 27%, 14% and 8% respectively. PDR at 14% and Particle Filters at 8% were also quite common. The proposed techniques use diverse sensor technologies, but IMUs are the most favoured at 57%, while camera sensors follow closely at 38%. Lidar sensors are preferred at 16%, and ultrasonic sensors at 14%. The popularity of IMU and camera sensors can be attributed to the widespread use of smartphones as the preferred platform for developing solutions for BVI individuals. Out of the proposed solutions, only 14% are effective in real-life situations, while 29% are practical but may have high costs or require multiple sensors for users to carry. Around 24% have limited practicality for specific scenarios, while 32% are purely experimental.

The second target was the comparison of commercial solutions assisting blind navigation in both outdoor and indoor spaces. The comparison study considered five applications, namely Blindsquare, Lazarillo, Ariadne GPS, Nav by ViaOpta and, finally, Seeing Assistant Move. It included a description of each application as well as a list of all the available features. Furthermore, part of the evaluation involved the Usability evaluation of Blindsquare, Lazarillo and our own application BlindRouteVision. Usability was evaluated with the metrics of Effectiveness, Efficiency and UX. The results of this study highlighted that the BVI individuals required a wealth of supported features from their application but were willing to give up to a certain extent several of them in the face of better and more precise navigation on corners and intersections, which constitute places of high risk during navigation.

On the other hand, the list highlighted the fact that all applications are utilizing the existing GPS technology to support outdoor navigation. A popular choice was the integration of external third-party services such as maps (Google Maps, Apple Maps, OpenStreetMaps and the like) for providing real-time navigation.

Furthermore, it made evident that a modern application targeting outdoor navigation for BVI individuals needs to have at minimum the following features. An easy way to search for places and start the navigation ideally with voice control or else have a screen reader compatible structure, accurate localization of the user’s position, real-time voice guidance, easily accessible way to regain both the current position and the direction as external events can throw off even the most experienced BVI individuals, route planning ahead of time, customizability of navigation instructions in terms of the wealth of information and format (orthogonal or clockwise) as cardinal instructions, commonly found in third-party navigation applications, are not useful as the cause of blindness, either congenital or acquired, shapes the BVI’s perception of the surrounding environment [1], more detailed navigation when crossing intersections, management of the frequency and priority of the emitted instructions as well as the support of predefined and user-defined POIs that can serve the role of navigational landmarks.

In order to significantly improve the provided quality of the solution, then the capability to filter other applications’ notifications and obstacle detection follow are much-needed features. When the navigation application is taking place, it should be prioritized over other applications and notifications from them should be limited. Other critical applications required by the user such as phone calls or messages should be added to a whitelist and become part of a priority hierarchy to resolve any potential conflicts. As far as obstacle detection is concerned, the existing commercial solutions do not support such functionality and, instead, it is performed manually by the BVI individual with the help of the white cane. Although all BVI individuals have acquired O&M skills allowing them to detect and avoid obstacles in their route, this is not always achieved easily or safely. A system that can proactively inform the users about impending obstacles, static and/or dynamic, can significantly improve the user’s effectiveness and efficiency and, thus, increase the rates of successfully reaching the destination. Between static and dynamic obstacle detection, the latter is more critical for the safety of the user as the white cane can be used to detect static objects while among the static objects critical to safety are the ones found on the user’s head level such as low hanging tree branches, low balconies, signs and the like. Another very useful feature is route simulation as it provides the opportunity for the user to better learn the route and the surrounding environment prior to traversing it.

Likewise to the previous case, a modern blind indoor navigation application needs to have at minimum the following. An easy way to search for POIs once the user has entered an indoor place, like emergency places, shops, toilet facilities and public spaces among others. At the very least it should support accurate localization of the user’s position, more fine-grained guidance in comparison to the outdoor case given the limited mobility and freedom of movement and error correction in case the user has stranded from the navigation path, real-time guidance, customizability of navigation instructions in terms of the wealth of information and frequency of emission, and an easy way for the user to access the current position as the stimulus of the external events may be overwhelming.

A significant improvement in blind indoor navigation can be accomplished by adding obstacle detection capabilities for both static and dynamic objects, and the provision of obstacle avoidance functionality. This is even more crucial for indoor spaces as quite often they are crowded, i.e., one can consider the example of a shopping mall, a hospital or a subway. Another improvement is the incorporation of beacon-based solutions such as the widely used BLE beacons. These beacons are placed at static places known to the application so as to minimize the accumulated error of the various localization techniques. Further improving the localization as well as the searching capabilities is the generation of digital maps specifically tailored to the requirements of the location. This can provide significant benefits; however, it requires the active participation of the people managing the place being mapped. The latter applies to both the scaling down of the infrastructure and to the ease of management of such dense information. Last but not least, is the use of simulation tools, to allow the user to create families of microprocessors and of other peripherals.

As far as it concerns the improvements of commercial applications, the scientific literature can provide new features for indoor and outdoor space navigation. For the issue of directionality due to the smartphone GPS sensors’ poor accuracy, external GPS receivers can be used as demonstrated in [7]. There the authors combine a high-accuracy external GPS receiver with a novel route navigation algorithm to achieve a better level of accuracy and guidance. For the obstacle detection feature many of the multi-sensor fusion solutions presented in the literature review can be leveraged. The main techniques employed include the use of ultrasonic sensors, computer vision and deep learning-based solutions. The use of cameras can significantly improve the commercially available applications; however, they do come with their own set of restrictions. Despite their undeniable benefits, camera-based solutions usually are associated with higher costs, as it is quite common to deploy more than one type of camera sensor and may incur an additional computational cost that may impact battery-based devices. Ultrasonic sensors, on the other hand, have a significantly lower cost on average and computational overhead but do not demonstrate the same strong results.

Despite the lack of sensor-based assistive solutions, the literature review demonstrated the benefits of this approach and an increasing trend in adopting these solutions, especially with computer vision and deep learning techniques as the main driver.

## Figures and Tables

**Table 1:** The techniques utilized in the research papers.

	Techniques	
Fuzzy Logic	Computer vision	Deep Learning
Pedestrian Dead Reckoning (PDR)	Monte Carlo	Kalman Filter
2D Object Detection	Semantic segmentation	2D Object Tracking
3D Point Cloud	Depth Maps	

**Table 2:** Type of sensors utilized in the research papers.

Sensors	Definition
Camera	Devices detecting and conveying information as an image
Inertial Measurement Unit (IMU)	A device consisting of gyroscopes, accelerometers and magnetometers measuring an object’s gravity and angular rate
RGB-D	Depth sensing devices that work with an RGB (Red, Green, Blue) sensor camera augmenting conventional images with depth information
Millimeter Wave	A special class of radar technology using short wavelength electromagnetic waves for object detection
Lidar	Laser-based devices that determine the ranges of obstacles in 3D space by measuring the time required for the reflected light to return to a receiver
Ultrasonic	Devices measuring object distance using ultrasonic sound waves
GPS	Satellite-based navigation system receivers that provide position, velocity and timing information
Gyroscope	Devices measuring angular velocity
Accelerometer	Devices that measure acceleration forces acting on an object to determine its position and movement.
Magnetometer	Devices measuring the magnetic field or the magnetic dipole moment
Beacon	Periodical transmission of information
Compass	Devices detecting and responding to the presence of a magnetic field
Microphone	Devices capturing sound waves and converting them into an electrical signal
Passive Infrared Resistor (PIR)	Devices measuring infrared (IR) light radiating from objects to detect motion
Vibration	Devices measuring vibration
Buzzer	An audio signalling device
Barometer	Devices measuring atmospheric pressure
Wi-Fi	Devices using Wi-Fi signals to transmit information.
Sonar	Devices measuring object distance using sonic sound waves
Thermal Camera	Devices creating images from infrared radiation instead of visible light

**Table 3:** Solutions and obstacle detection.

Paper	Support	Static	Dynamic	Range	Accuracy
[41,42,43,44,45,46,47]	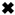	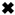	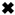	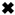	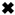
[48]	✅	**N/A**	**N/A**	**N/A**	**N/A**
[49]	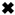	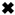	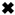	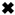	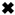
[50]	✅	**N/A**	**N/A**	**N/A**	**N/A**
[51]	✅	**N/A**	**N/A**	**5 m**	**95%**
[52]	✅	✅	✅	**0 m < R < 9 m**	**98%**
[53]	✅	✅	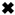	**0.1 m < R < 3.5 m**	**90–95%**
[54]	✅	**N/A**	**N/A**	**N/A**	**N/A**
[55]	✅	**N/A**	**N/A**	**0.2 m < R < 10 m**	**N/A**
[56]	✅	✅	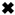	**R > 2 m**	**N/A**
[57]	✅	✅	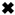	**N/A**	**N/A**
[58]	✅	✅	✅	**2 cm < R < 4.5 m**	**N/A**
[59]	✅	✅	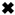	**N/A**	**67–98%**
[60]	✅	✅	✅	**2 cm < R < 12 m**	**N/A**
[61,62,63]	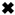	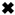	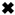	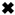	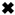
[64]	✅	✅	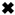	**R = 20 cm**	**N/A**
[65,66,67,68,69,70,71]	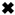	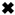	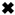	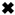	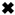
[72]	✅	✅	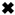	**N/A**	**N/A**
[73]	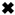	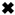	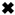	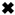	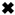
[74]	✅	✅	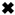	**R < 6 m**	**N/A**
[75,76,77,78]	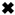	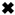	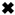	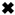	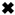

**Table 4:** Commercial applications and features.

	Blindsquare	Lazarillo	Nav by ViaOpta	Ariadne GPS	Seeing Assistant Move
Platform	iOS	Android & iOS	Android & iOS	iOS	Android & iOS
Indoor navigation	✅	✅	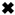	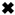	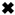
Outdoor navigation	✅	✅	✅	✅	✅
Real-time update	✅	✅	✅	✅	✅
Report the user’s location	✅	✅	✅	✅	✅
Look around	✅	✅	✅	✅	✅
Creation of digital maps	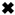	✅	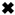	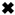	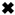
Intersections	✅	✅	✅	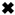	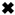
Simulation mode	✅	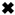	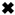	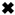	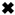
3rd party app navigation	✅	✅	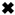	✅	✅
Voice control	✅	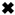	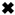	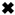	✅
Supports deafblind	✅	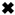	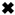	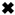	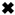
Search places	✅	✅	✅	✅	✅
Different input modes	✅	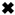	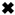	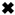	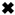
Audio menu	✅	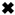	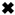	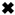	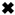
POIs	✅	✅	✅	✅	✅
Adjustable radius—POIs	✅	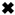	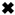	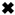	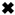
Filtering POIS	✅	✅	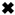	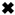	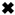
Alert Distance	✅	✅	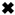	✅	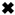
Public transport information	✅	✅	✅	✅	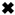
Vehicle mode	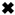	✅	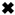	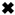	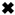
Customizable	✅	✅	✅	✅	✅
Built-in turn by turn	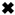	✅	✅	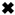	✅
Increasing frequency proximity progress updates	✅	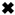	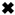	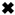	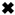
Repeating information	✅	✅	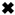	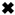	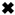
Cardinal directions	✅	✅	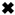	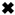	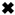
Multi-category search	✅	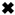	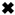	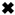	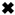
Sharing places	✅	✅	✅	✅	✅
Call a sighted person for live support	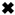	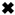	✅	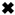	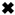
Route planning	✅	✅	✅	✅	✅
Total	25	19	13	12	12

**Table 5:** Effectiveness and efficiency results.

Applications	Effectiveness	Efficiency
BlindRouteVision	80.56%	78.12%
Blindsquare	78%	73%
Lazarillo	66.6%	62%

## Data Availability

Not applicable.
